# Complete genome sequence of the facultatively chemolithoautotrophic and methylotrophic alpha Proteobacterium *Starkeya novella* type strain (ATCC 8093^T^)

**DOI:** 10.4056/sigs.3006378

**Published:** 2012-09-26

**Authors:** Ulrike Kappler, Karen Davenport, Scott Beatson, Susan Lucas, Alla Lapidus, Alex Copeland, Kerrie W. Berry, Tijana Glavina Del Rio, Nancy Hammon, Eileen Dalin, Hope Tice, Sam Pitluck, Paul Richardson, David Bruce, Lynne A. Goodwin, Cliff Han, Roxanne Tapia, John C. Detter, Yun-juan Chang, Cynthia D. Jeffries, Miriam Land, Loren Hauser, Nikos C. Kyrpides, Markus Göker, Natalia Ivanova, Hans-Peter Klenk, Tanja Woyke

**Affiliations:** 1 The University of Queensland, Brisbane, Australia; 2 Los Alamos National Laboratory, Bioscience Division, Los Alamos, New Mexico, USA; 3 DOE Joint Genome Institute, Walnut Creek, California, USA; 4 Oak Ridge National Laboratory, Oak Ridge, Tennessee, USA; 5 Leibniz Institute DSMZ – German Collection of Microorganisms and Cell Cultures, Braunschweig, Germany

**Keywords:** strictly aerobic, facultatively chemoautotrophic, methylotrophic and heterotrophic, Gram-negative, rod-shaped, non-motile, soil bacterium, *Xanthobacteraceae*, CSP 2008

## Abstract

*Starkeya novella* (Starkey 1934) Kelly *et al*. 2000 is a member of the family *Xanthobacteraceae* in the order *‘Rhizobiales’*, which is thus far poorly characterized at the genome level. Cultures from this species are most interesting due to their facultatively chemolithoautotrophic lifestyle, which allows them to both consume carbon dioxide and to produce it. This feature makes *S. novella* an interesting model organism for studying the genomic basis of regulatory networks required for the switch between consumption and production of carbon dioxide, a key component of the global carbon cycle. In addition, *S. novella* is of interest for its ability to grow on various inorganic sulfur compounds and several C1-compounds such as methanol. Besides *Azorhizobium caulinodans*, *S. novella* is only the second species in the family *Xanthobacteraceae* with a completely sequenced genome of a type strain. The current taxonomic classification of this group is in significant conflict with the 16S rRNA data. The genomic data indicate that the physiological capabilities of the organism might have been underestimated. The 4,765,023 bp long chromosome with its 4,511 protein-coding and 52 RNA genes was sequenced as part of the DOE Joint Genome Institute Community Sequencing Program (CSP) 2008.

## Introduction

Strain ATCC 8093^T^ (ATCC 8093 = DSM 506 = NBRC 14993) is the type strain of the species *Starkeya novella* [1] and the type species of the genus *Starkeya* [1], which currently contains only one other species, *S. koreensis* [2]. The most prominent feature of *S. novella* is its ability to grow as a facultative chemolithoautotroph [3], a heterotroph [4], or methylotroph [1,5]. Cultures of strain ATCC 8093^T^ were first isolated from soil samples taken from agricultural land in New Jersey by Robert L. Starkey in the early 1930s [6,7] and deposited in the American Type Culture Collection (ATCC) under the basonym *Thiobacillus novellus* [3,8]. The bacterium was referred to as the ‘new’ *Thiobacillus* as it was the first facultatively chemolithoautotrophic sulfur oxidizer to be isolated. Until then, all known dissimilatory sulfur-oxidizing bacteria were also obligate autotrophs. As a result, the metabolism of *T. novellus* was intensely studied for many years following its discovery, and particularly following the development of more sophisticated biochemical and molecular methods in the 1960s.

During the last fifty years, the strain has been used in numerous molecular studies, both of its oxidative sulfur metabolism and the versatility and regulation of its carbon metabolism. Studies included generation of reducing power in chemosynthesis [9], carbon dioxide fixation and carboxydismutase action [10], catabolite repression in facultative chemoautotrophs [11], regulation of glucose transport and metabolism [12], isolation and characterization of a bacteriophage [13], pathways of thiosulfate oxidation [9,14-17], the formation of sulfite during the oxidation of thiosulfate [18], and the isolation and characterization of a bacterial sulfite dehydrogenase [19-29], a sulfite-oxidizing enzyme.

Based on the 16S rRNA gene sequence in 2000 Kelly *et al.* [1] proposed the reclassification of *T. novellus* to *S. novella*. The genus name *Starkeya* is in honor of Robert L. Starkey and his important contribution to soil microbiology and sulfur biochemistry [1]; the species epithet was derived from the Latin adjective ‘*novella*’, new [3]. Here we present a summary classification and a set of features for *S. novella* ATCC 8093^T^, together with the description of the genomic sequencing and annotation.

## Classification and features

### 16S rRNA analysis

The single genomic 16S rRNA sequence of strain ATCC 8093T was compared using NCBI BLAST [30,31] under default settings (e.g., considering only the high-scoring segment pairs (HSPs) from the best 250 hits) with the most recent release of the Greengenes database [32] and the relative frequencies of taxa and keywords (reduced to their stem [33]) were determined, weighted by BLAST scores. The most frequently occurring genera were *Ancylobacter* (30.0%), *Starkeya* (13.4%), *Agrobacterium* (13.1%), *Xanthobacter* (12.4%) and *Azorhizobium* (11.5%) (98 hits in total). Regarding the three hits to sequences from members of the species, the average identity within HSPs was 99.5%, whereas the average coverage by HSPs was 92.8%. Among all other species, the one yielding the highest score was *Ancylobacter rudongensis* (AY056830), which corresponded to an identity of 98.1% and an HSP coverage of 98.4%. (Note that the Greengenes database uses the INSDC (= EMBL/NCBI/DDBJ) annotation, which is not an authoritative source for nomenclature or classification.) The highest-scoring environmental sequence was EU835464 ('structure and quorum sensing reverse osmosis RO membrane biofilm clone 3M02'), which showed an identity of 98.4% and an HSP coverage of 100.0%. The most frequently occurring keywords within the labels of all environmental samples which yielded hits were 'skin' (6.0%), 'microbiom' (3.0%), 'human, tempor, topograph' (2.5%), 'compost' (2.1%) and 'dure' (2.1%) (152 hits in total) and fit only partially to the known habitat of the species. Environmental samples that yielded hits of a higher score than the highest scoring species were not found.

**Figure 1:**
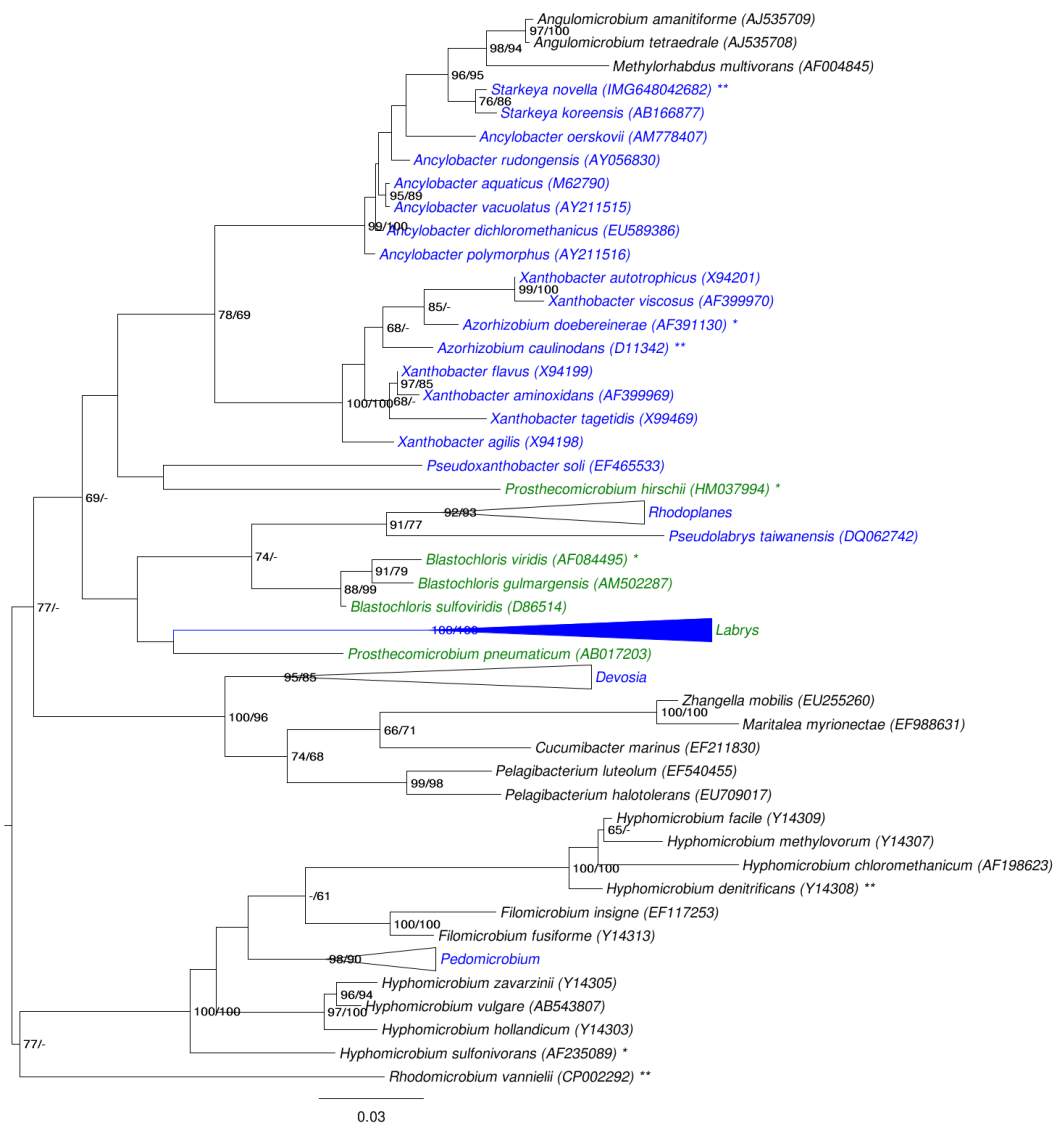
Phylogenetic tree highlighting the position of *S. novella* relative to the type strains of the other species within the family *Xanthobacteraceae* (blue font color). The tree was inferred from 1,381 aligned characters [34,35] of the 16S rRNA gene sequence under the maximum likelihood (ML) criterion [36]. *Hyphomicrobiaceae* (green font color for those species that caused conflict according to the Parafit test, black color for the remaining ones; see below for the difference) were included in the dataset for use as outgroup taxa but then turned out to be intermixed with the target family; hence, the rooting shown was inferred by the midpoint-rooting method [29]. The branches are scaled in terms of the expected number of substitutions per site. Numbers adjacent to the branches are support values from 550 ML bootstrap replicates [37] (left) and from 1,000 maximum-parsimony bootstrap replicates [38] (right) if larger than 60%. Lineages with type strain genome sequencing projects registered in GOLD [39] are labeled with one asterisk, those also listed as 'Complete and Published' with two asterisks (see [40] and CP000781 for *Xanthobacter autotrophicus*, CP002083 for *Hyphomicrobium denitrificans* and CP002292 for *Rhodomicrobium vannielii*).

Figure 1 shows the phylogenetic neighborhood of in a 16S rRNA based tree. The sequence of the single 16S rRNA gene copy in the genome differs by nine nucleotides from the previously published 16S rRNA sequence (D32247), which contains one ambiguous base call.

To measure conflict between 16S rRNA data and taxonomic classification in detail, we followed a constraint-based approach as described recently in detail [41], conducting both unconstrained searches and searches constrained for the monophyly of both families and using our own re-implementation of CopyCat [42] in conjunction with AxPcoords and AxParafit [43] was used to determine those leaves (species) whose placement significantly deviated between the constrained and the unconstrained tree.

The best-supported ML tree had a log likelihood of -12,191.55, whereas the best tree found under the constraint had a log likelihood of -12,329.92. The constrained tree was significantly worse than the globally best one in the SH test as implemented in RAxML [37,44] (α = 0.01). The best supported MP trees had a score of 1,926, whereas the best constrained trees found had a score of 1.982 and were also significantly worse in the KH test as implemented in PAUP [8,44] (α < 0.0001). Accordingly, the current classification of the family as used in [45,46], on which the annotation of Figure 1 is based, is in significant conflict with the 16S rRNA data. Figure 1 also shows those species that cause phylogenetic conflict as detected using the ParaFit test (i.e., those with a p value > 0.05 because ParaFit measures the significance of congruence) in green font color. According to our analyses, the *Hyphomonadaceae* genera (*Blastochloris* and *Prosthecomicrobium*) nested within the *Xanthobacteraceae* display significant conflict. In the constrained tree (data not shown), the *Angulomicrobium-Methylorhabdus* clade is placed at the base of the *Xanthobacteraceae* clade (forced to be monophyletic). For this reason, *Angulomicrobium* and *Methylorhabdus* were not detected as causing conflict (note that the ParaFit test essentially compares unrooted trees). A taxonomic revision of the group would probably need to start with the reassignment of these genera to different families.

### Morphology and physiology

**Figure 2:**
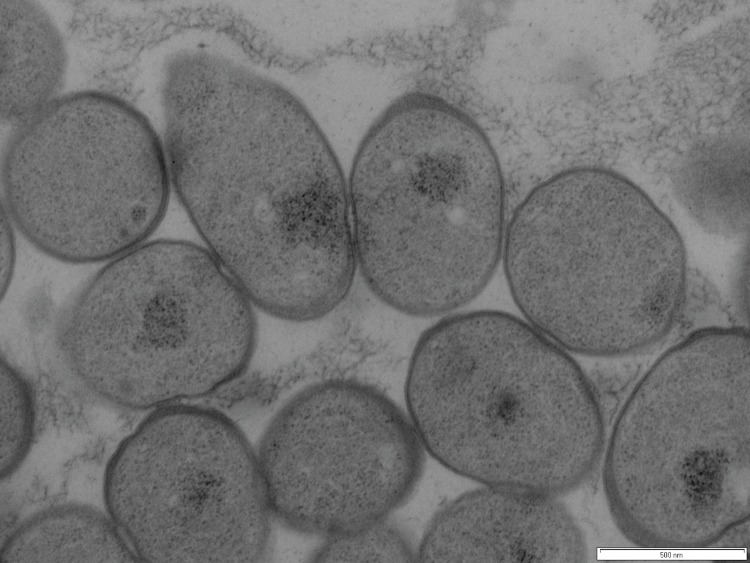
Transmission electron micrograph of *S. novella* ATCC 8093^T^. Scale bar: 500 nm

Cells of *S. novella* ATCC 8093^T^ are non-motile, Gram-negative staining short rods or coccobacilli with a size of 0.4–0.8 μm × 0.8–2.0 μm, occurring singly or in pairs (Figure 2, Table 1) [1]. Colonies grown on thiosulfate agar turn white with sulfur on biotin supplemented growth media [1], while in the presence of small amounts of yeast extract (DSMZ medium 69) the colonies have a pale pink appearance following growth on thiosulfate and no sulfur formation is observed. Cells grow on thiosulfate and tetrathionate under aerobic conditions, but not on sulfur or thiocyanate [1]. Ammonium salts, nitrates, urea and glutamate can serve as nitrogen sources [1]. Several surveys of substrates supporting heterotrophic growth have been published, and include glucose, formate, methanol, oxalate [1,2,4,6]. The growth range spans from 10-37°C, with an optimum at 25-30°C, and a pH range from 5.7-9.0 with an optimum at pH 7.0 [1].

**Table 1:** Classification and general features of *S. novella* according to the MIGS recommendations [47] and the NamesforLife database [48].

**MIGS ID**	**Property**	**Term**	**Evidence code**
	Current classification	Domain *Bacteria*	TAS [49]
		Phylum *Proteobacteria*	TAS [50]
		Class *Alphaproteobacteria*	TAS [51,52]
		Order *‘Rhizobiales’*	TAS [52,53]
		Family *Xanthobacteraceae*	TAS [54]
		Genus *Starkeya*	TAS [1]
		Species *Starkeya novella*	TAS [1]
		Type strain ATCC 8093	TAS [1]
	Gram stain	negative	TAS [1]
	Cell shape	rod-shaped (some coccobacilli)	TAS [1]
	Motility	non-motile	TAS [1]
	Sporulation	not reported	
	Temperature range	mesophile, 10–37°C	TAS [1]
	Optimum temperature	25–30°C	TAS [1]
	Salinity	not reported	
MIGS-22	Oxygen requirement	strictly aerobic	TAS [1]
	Carbon source	CO_2_, citrate, glutamic acid (among others)	TAS [1,3]
	Energy metabolism	facultatively chemolithoautotroph and methylotroph, heterotroph	TAS [1,5]
MIGS-6	Habitat	soil	TAS [1]
MIGS-15	Biotic relationship	free living	NAS
MIGS-14	Pathogenicity	none	NAS
	Biosafety level	1	TAS [55]
MIGS-23.1	Isolation	soil	TAS [1]
MIGS-4	Geographic location	not reported (probably New Jersey)	
MIGS-5	Sample collection time	1934 or before	TAS [6,7]
MIGS-4.1	Latitude	not reported	
MIGS-4.2	Longitude	not reported	
MIGS-4.3	Depth	not reported	
MIGS-4.4	Altitude	not reported	

Evidence codes - TAS: Traceable Author Statement (i.e., a direct report exists in the literature); NAS: Non-traceable Author Statement (i.e., not directly observed for the living, isolated sample, but based on a generally accepted property for the species, or anecdotal evidence). Evidence codes are from the Gene Ontology project [56].

### Chemotaxonomy

The lipopolysaccharide of strain ATCC 8093^T^ lacks heptoses and has only 2,3-diamino-2,3-dideoxyglucose as the backbone sugar [1]; other data on the cell wall structure of strain ATCC 8093^T^ are not available. The major isoprenoid quinone is ubiquinone Q-10 [1], and the major cellular fatty acids are octadecenoid acid (C_18:1_) and C_19_ cyclopropane acid; no hydroxyl acids are present [1]. Cells contain putrescine and homospermidine.

## Genome sequencing and annotation

### Genome project history

This organism was selected for sequencing on the basis of the DOE Joint Genome Institute Community Sequencing Program (CSP) 2008. The genome project is deposited in the Genomes On Line Database [39] and the complete genome sequence is deposited in GenBank. Sequencing, finishing and annotation were performed by the DOE Joint Genome Institute (JGI). A summary of the project information is shown in Table 2.

**Table 2:** Genome sequencing project information

**MIGS ID**	**Property**	**Term**
MIGS-31	Finishing quality	Finished
MIGS-28	Libraries used	Three genomic libraries: one 454 pyrosequence standard library, one 454 PE library (22 kb insert size), one Illumina library
MIGS-29	Sequencing platforms	Illumina GAii, 454 GS FLX Titanium
MIGS-31.2	Sequencing coverage	44.3 × Illumina; 53.5 × pyrosequence
MIGS-30	Assemblers	Newbler version 2.0.1-PreRelease-03-30-2009, Velvet, phrap version SPS - 4.24
MIGS-32	Gene calling method	Prodigal
	INSDC ID	CP002026
	GenBank Date of Release	November 21, 2011
	GOLD ID	Gc01353
	NCBI project ID	37659
	Database: IMG-GEBA	648028054
MIGS-13	Source material identifier	DSM 506
	Project relevance	Carbon cycle, Environmental

### Growth conditions and DNA isolation

Strain ATCC 8093^T^ was grown from a culture of DSMZ 506 in DSMZ medium 69 at 28°Cg DNA was purified using the Genomic-tip 100 System (Qiagen) following the directions provided by the supplier. The purity, quality and size of the bulk gDNA preparation were assessed by JGI according to DOE-JGI guidelines.

### Genome sequencing and assembly

The genome was sequenced using a combination of Illumina and 454 sequencing platforms. All general aspects of library construction and sequencing can be found at the JGI website [57]. Pyrosequencing reads were assembled using the Newbler assembler (Roche). The initial Newbler assembly consisting of 13 contigs in one scaffold was converted into a phrap [58] assembly by making fake reads from the consensus, to collect the read pairs in the 454 paired end library. Illumina GAii sequencing data (211.3 Mb) were assembled with Velvet [59] and the consensus sequences were shredded into 1.5 kb overlapped fake reads and assembled together with the 454 data. The 454 draft assembly was based on 259.9 Mb 454 draft data and all of the 454 paired-end data. Newbler parameters were -consed -a 50 -l 350 -g -m -ml 20. The Phred/Phrap/Consed software package [58] was used for sequence assembly and quality assessment in the subsequent finishing process. After the shotgun stage, reads were assembled with parallel phrap (High Performance Software, LLC). Possible mis-assemblies were corrected with gapResolution [58], Dupfinisher [60], or sequencing cloned bridging PCR fragments with subcloning. Gaps between contigs were closed by editing in Consed, by PCR and by Bubble PCR primer walks (J.-F. Chang, unpublished). A total of 43 additional reactions were necessary to close gaps and to raise the quality of the finished sequence. Illumina reads were also used to correct potential base errors and increase consensus quality using a software Polisher developed at JGI [61]. The error rate of the completed genome sequence is less than 1 in 100,000. Together, the combination of the Illumina and 454 sequencing platforms provided 97.8 × coverage of the genome. The final assembly contained 865,253 pyrosequence and 6,036,863 Illumina reads.

### Genome annotation

Genes were identified using Prodigal [62] as part of the Oak Ridge National Laboratory genome annotation pipeline, followed by a round of manual curation using the JGI GenePRIMP pipeline [63]. The predicted CDSs were translated and used to search the National Center for Biotechnology Information (NCBI) non-redundant database, UniProt, TIGRFam, Pfam, PRIAM, KEGG, COG, and InterPro databases. These data sources were combined to assert a product description for each predicted protein. Non-coding genes and miscellaneous features were predicted using tRNAscan-SE [64, RNAMMer [65], Rfam [66], TMHMM [67], and SignalP [68].

## Genome properties

**Figure 3:**
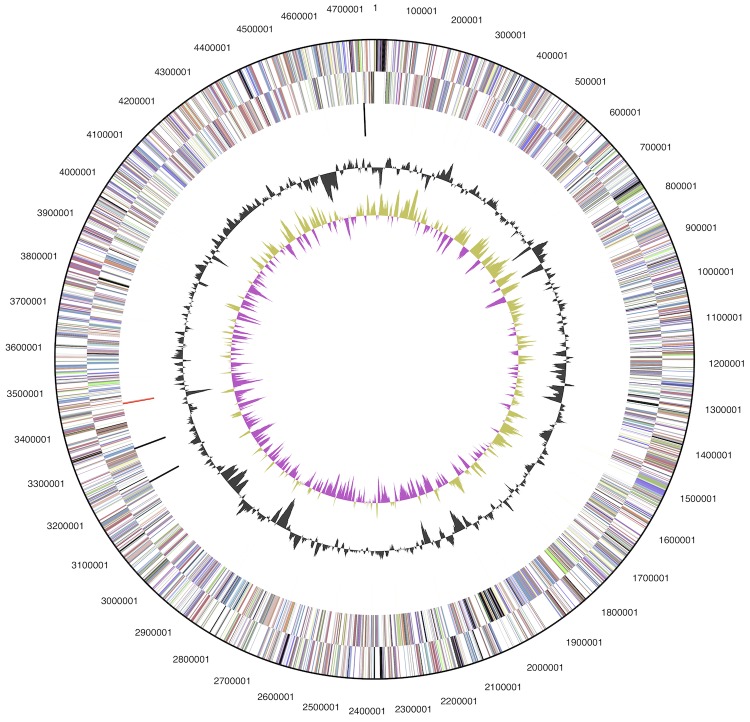
Graphical map of the chromosome. From outside to the center: Genes on forward strand (color by COG categories), Genes on reverse strand (color by COG categories), RNA genes (tRNAs green, rRNAs red, other RNAs black), GC content (black), GC skew (purple/olive).

The genome consists of a circular 4,765,023 bp chromosome a 67.9% G+C content (Table 3 and Figure 3). Of the 4,563 genes predicted, 4,511 were protein-coding genes, and 52 RNAs; 80 pseudogenes were also identified. The majority of the protein-coding genes (74.8%) were assigned a putative function while the remaining ones were annotated as hypothetical proteins. The distribution of genes into COGs functional categories is presented in Table 4. A total of 388 genes are predicted to encode proteins involved in signal transduction, including 284 one-component systems, 41 histidine kinases, 47 response regulators, seven chemotaxis proteins and two additional unclassified proteins.

**Table 3:** Genome Statistics

**Attribute**	**Value**	**% of Total**
Genome size (bp)	4,765,023	100.00%
DNA coding region (bp)	4,222,317	88.61%
DNA G+C content (bp)	3,234,723	67.88%
Number of replicons	1	
Extrachromosomal elements	0	
Total genes	4,563	100.00%
RNA genes	52	1.14%
rRNA operons	1	
tRNA genes	46	1.01%
Protein-coding genes	4,511	98.86%
Pseudo genes	80	1.75%
Genes with function prediction (proteins)	3,413	74.80%
Genes in paralog clusters	2,690	58.95%
Genes assigned to COGs	3,582	78.50%
Genes assigned Pfam domains	3,730	81.74%
Genes with signal peptides	1,730	37.91%
Genes with transmembrane helices	1,169	25.62%
CRISPR repeats	0	

**Table 4:** Number of genes associated with the general COG functional categories

**Code**	**value**	**% age**	**Description**
J	176	4.5	Translation, ribosomal structure and biogenesis
A	0	0.0	RNA processing and modification
K	303	7.7	Transcription
L	118	3.0	Replication, recombination and repair
B	2	0.1	Chromatin structure and dynamics
D	30	0.8	Cell cycle control, cell division, chromosome partitioning
Y	0	0.0	Nuclear structure
V	54	1.4	Defense mechanisms
T	181	4.6	Signal transduction mechanisms
M	210	5.3	Cell wall/membrane biogenesis
N	8	0.2	Cell motility
Z	0	0.0	Cytoskeleton
W	0	0.0	Extracellular structures
U	36	0.9	Intracellular trafficking and secretion, and vesicular transport
O	148	3.8	Posttranslational modification, protein turnover, chaperones
C	291	7.4	Energy production and conversion
G	270	6.9	Carbohydrate transport and metabolism
E	504	12.8	Amino acid transport and metabolism
F	77	2.0	Nucleotide transport and metabolism
H	156	4.0	Coenzyme transport and metabolism
I	143	3.6	Lipid transport and metabolism
P	229	5.8	Inorganic ion transport and metabolism
Q	105	2.7	Secondary metabolites biosynthesis, transport and catabolism
R	487	12.4	General function prediction only
S	405	10.3	Function unknown
-	981	21.5	Not in COGs

## Insights into the genome

As indicated in the introduction, because *S. novella* was the first facultative sulfur chemolithotrophic bacterium to be isolated, many studies of its metabolic capabilities were carried out following its discovery. Several groups worked on the carbon metabolism of *S. novella*, which led to the discovery of an operational pentose phosphate pathway in this bacterium [69], which is also the only reported pathway of glucose metabolism in the description of *S. novella* [1]. However, analysis of the genome sequence revealed that in addition to a pentose phosphate pathway, *S. novella* also contains enzymes required for the Entner-Doudoroff pathway (Snov_2999 & Snov_3400, 2-dehydro-3-deoxyphosphogluconate aldolase; 6-phosphogluconate dehydratase; biocyc database) and the enzymes required for the Embden-Meyerhoff pathway, although this pathway appears to lack a phosphofructokinase (EC 2.7.1.11), indicating that it may only be able to be used for gluconeogenesis.

The respiratory chain of *S. novella* has also been studied and an aa_3_ type terminal oxidase was identified and characterized in some detail [70-73]. It was also discovered that the cytochrome *c* that interacts with this cytochrome oxidase (most likely this cytochrome is encoded by Snov_1033) has properties that are reminiscent of the mitochondrial respiratory chain cytochrome *c* [70-75], including a high pI and an ability to transfer electrons to the bovine cytochrome oxidase [76]. The analysis of the genome revealed a much greater diversity of respiratory chain complexes than previously recognized, including two NADH oxidases (gene regions Snov_1853 & Snov_2407), one succinate dehydrogenase (Snov_3317 gene region) and a cytochrome *bc*_1_ complex (Snov_2477 gene region). In addition to these components, the genome encodes two aa_3_ type cytochrome oxidases (gene regions Snov_0584 & 4240), two cytochrome bd type quinol oxidases (pfam02322, gene regions Snov_0620 & 3535), a cbb_3_ type cytochrome oxidase (gene region Snov_4464), and a cyoB type quinol oxidase (COG0843, cd01662, gene region Snov_1015) indicating a significant versatility of respiration in *S. novella* as well as the potential to grow at low oxygen tensions as both the cbb_3_ and bd type oxidases are known to have high affinities for oxygen, enabling growth under microaerophilic conditions. Experiments in our laboratory have shown that final OD_600_ values reached by cultures grown on thiosulfate (5g/l) and hydrogen carbonate (20 mM) supplemented DSMZ medium 69 were the same regardless of whether 25, 50, 100 or 200 ml of medium were used in a 250 ml flask. This clearly confirms that, as indicated by the genome data, *S. novella* is capable of growth under microaerophilic as well as aerobic conditions.

We also re-evaluated the range of substrates that support growth of *S. novella*. In the description of the genus *Starkeya* [1] only glucose, formate, methanol and oxalate were listed as growth-supporting substrates in addition to thiosulfate and tetrathionate. An early paper reporting a test of the heterotrophic potential of *S. novella* was published in 1969 by Taylor and Hoare [4] in which they identified 16 potential growth substrates (Table no. 7 in [4]) including all of the above except oxalate, which was identified subsequently by [5] who were seeking to evaluate the C_1_ compound metabolism of *S. novella* and also identified formamide as a potential substrate. It is unclear why the description of the genus *Starkeya* did not list all of the 16 growth substrates identified by Taylor and Hoare. To confirm the earlier data, we carried out a growth substrate screen using the Biolog system (GN2 assay plates) as well as an api20NE test for bacterial identification. Some substrates that are not part of this Biolog GN2 plate (e.g. oxalate, fructose, succinate etc.) were independently tested in the laboratory for their ability to support growth. In the API20NE test, in addition to a positive oxidase response, *S. novella* tested positive for ESC/Fecit and p-nitrophenyl hydrolysis, glucose, mannitol and gluconate utilization. The Biolog assay clearly showed that the heterotrophic potential of this bacterium is greater than previously identified, with a total of 28 growth-supporting substrates being identified in the screen (Table 5). The metabolic profile could not be identified as such, and was most closely related to that of *Ancylobacter aquaticus* (SIM: 0.45, Dist: 8.96), which supports the phylogenetic placement of *S. novella* in the *Ancylobacter* subgroup of the *Xanthobacteriaceae*. When combining all the data from the various studies, there are now 39 substrates that have been identified as supporting heterotrophic growth of *S. novella*. In addition to sugars such as glucose, fructose and arabinose, several sugar alcohols and amino acids as well as some organic acids can be used as growth substrates (Table 5). This reasonably large range of growth substrates is reflected in the size and the diversity of metabolic pathways present in the *S. novella* genome which, with a size of 4.6 Mb, is comparable to the genomes of e.g., *Escherichia coli* and *Rhodopseudomonas palustris*.

**Table 5:** Growth substrates utilized by *S. novella*

**Substrate**		**Substrate**	
D-glucose	+	*L-Histidine*	*+*
D-fructose	+	Proline	+
Sucrose	-	l-Leucine	-
D-Galactose	+	L-Isoleucine	-
L-arabinose	+	L-Tryptophan	-
D-gluconate	+	DL-Serine	+
D-arabitol	+	D-alanine	(+)
Adonitol	+	L-alanine	-
Xylitol	+	L-Glutamate	-
D-sorbitol	+	L-threonine	+
D-Mannitol	+	L-aspartate	-
Lactose	-	hydroxy-L Proline	+
*Maltose*	***+***	L-Alaninamide	+
D-Ribose	(+)	DL- Lactate	+
Glycerol	+	Malate	-
Pyruvate	+	Succinate	(+)
Formate	+	Fumarate	-
Formamide	+	Citrate	-
Formaldehyde	-	Methylpyruvate	+
Methylamine	-	Monomethylsuccinate	+
Trimethylamine	-	Alpha ketobutyrate	+
H2/CO2	-	Alpha hydroxybutyrate	+
Ethylamine	-	Beta hydroxy butyrate	+
Oxalate	+	Gamma aminobutyrate	+
Acetate	+	Benzoate	-
Propionate	+	p-Hydroxybenzoate	-
Butyrate	-	m-Hydroxybenzoate	-
Methanol	+	p-Aminobenzoate	-
Ethyl alcohol	+	Cyclohexanol	-
n-Propanol n-Butyl alcohol	+ -	Cyclohexane carboxylate	-
			

Results are combined from work done for this paper and [4-6]+ = substrate utilized, - = substrate not utilized, (+) = weak growth supported or ambiguous results in growth tests, italics = different results obtained in growth studies by different authors.

Although the analyses presented above are limited, they clearly illustrate that while the genome data confirm many of the results from early studies of the physiology of this bacterium, the metabolic capabilities of *S. novella* as indicated by the genome data clearly exceed those previously published in the literature and suggest that the versatility and adaptability to changing environments likely is a significant factor for its survival.
